# General Anesthetics Regulate Autophagy via Modulating the Inositol 1,4,5-Trisphosphate Receptor: Implications for Dual Effects of Cytoprotection and Cytotoxicity

**DOI:** 10.1038/s41598-017-11607-0

**Published:** 2017-09-28

**Authors:** Gongyi Ren, Yachun Zhou, Ge Liang, Bin Yang, Meirong Yang, Alexander King, Huafeng Wei

**Affiliations:** 1 Department of Anesthesiology and Critical Care, Perelman School of Medicine, University of Pennsylvania, Philadelphia, PA 19104 USA; 2 Department of Anesthesiology, Shanghai General Hospital, Shanghai Jiaotong University, Shanghai, China

## Abstract

General anesthetics are both neuroprotective and neurotoxic with unclear mechanisms. General anesthetics may control cell survival via their effects on autophagy by activation of type 1 inositol triphosphate receptor (InsP_3_R-1). DT40 or SH-SY5Y cells with only or over 99% expression of InsP_3_R-1 were treated with isoflurane or propofol. Cell viability was determined by MTT reduction or LDH release assays. Apoptosis was determined by measuring Caspase-3 or by TUNEL assay. Autophagy activity was determined by measuring LC3 II and P62. We evaluated mitochondrial integrity using MitoTracker Green and cytosolic ATP levels. Fura2-AM was used to measure the concentrations of cytosolic calcium ([Ca^2+^]_c_). Propofol significantly increased peak and integrated calcium response (P < 0.001) in cells with InsP_3_R-1 but not in cells with triple knockout of InsP_3_R. Both propofol and isoflurane increased autophagy induction (P < 0.05) in an mTOR- and InsP_3_R- activity dependent manner. Short exposure to propofol adequately activated InsP_3_-1 to provide sufficient autophagy for cytoprotection, while prolonged exposure to propofol induced cell apoptosis via impairment of autophagy flux through over activation of InsP_3_-1. Propofol damaged mitochondria and decreased cytosolic ATP. The effects of general anesthetics on apoptosis and autophagy are closely integrated; both are caused by differential activation of the type 1 InsP_3_R.

## Introduction

General anesthetics can be cardiac- or neuro-protective agents^1,2^. However, general anesthetics can also cause neuronal injury or death associated with behavioral and developmental abnormalities^3^. Intracellular Ca^2+^ homeostasis is important in maintaining cell survival and its disruption can result in cell damage^4^. Various GAs have demonstrated the ability to affect intracellular Ca^2+^ homeostasis via the InsP_3_ receptor (InsP_3_R) located on the membrane of the endoplasmic reticulum (ER). Among the three subtypes of InsP_3_R, the type-1 InsP_3_Rs (InsP_3_R-1) play important roles in many pathological conditions, including apoptosis, neuronal injury or death, ischemia and cell toxicity.

Autophagy is an adaptive survival response by cells to various stresses^5^. It is regulated by a number of protein kinases, with the mammalian target of rapamycin (mTOR) the best characterized^6^. Intracellular Ca^2+^ homeostasis, especially when regulated through activation of InsP_3_R, plays a crucial role in regulating autophagy. Ca^2+^ release from the ER via InsP_3_Rs elevates cytoplasmic Ca^2+^ concentration ([Ca^2+^]_i_) and activates autophagy via a mTOR-dependent pathway^6^. Considering InsP_3_R’s regulatory role in autophagy and the capacity of GAs to activate InsP_3_R^7^, it is reasonable to propose that GAs may control cell survival fate by affecting autophagy via differential activation of InsP_3_R.

Interestingly, newborn developing brains in various animal models and in some clinical studies have demonstrated a high sensitivity and vulnerability to anesthetic neurotoxicity^8^, with an unclear mechanism. One proposed mechanism is the disruption of intracellular Ca^2+^ homeostasis via excessive activation of InsP_3_R or ryanodine receptors (RYR)^9^. Notably, the expression of InsP_3_R may be associated with the maturation of neurons^10^. Apoptosis may also be significantly different between immature neurons and mature neurons because of differing autophagy regulation^11^. It is possible that differential effects of GAs on autophagy via activation of InsP_3_R may account for the high vulnerability of the developing brain to GA-mediated neuronal injury or death.

In various cell culture models, we have demonstrated that low concentrations of isoflurane or propofol for short durations stimulate cytoprotective autophagy by an mTOR-dependent pathway via adequate activation of InsP_3_R-1. However, GAs at high concentrations for prolonged use can impair autophagy flux and cause cell death via over-activation of InsP_3_R-1. Our study discloses a novel molecular mechanism for GAs’ dual effects of cytoprotection and cytotoxicity, which may provide a basis for better utilization of GAs.

## Results

### Anesthetic-induced autophagy via mTOR-dependent pathway by activation of type 1 InsP_3_R

**Figure 1:**
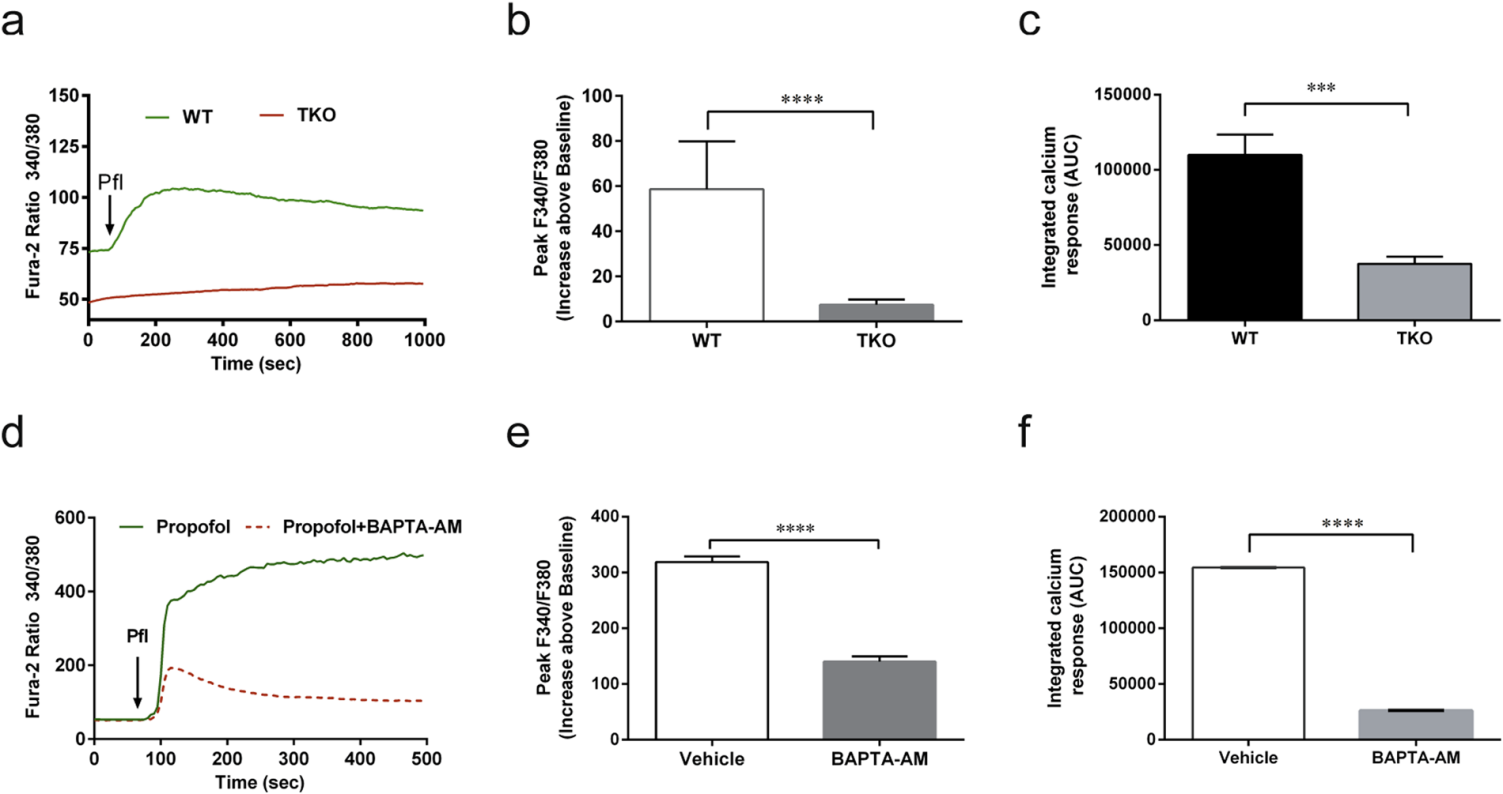
Propofol induced Ca^2+^ release from the ER via activation of InsP_3_R. (**a**) Typical response of propofol (pfl)-mediated elevation of cytosolic Ca^2+^ concentration ([Ca^2+^]_c_), which was indirectly reflected by measuring the 340/380 ratio using the Ca^2+^ indicator dye Fura-2 AM in DT40 WT or TKO cells (**a–c**) or SH-SY5Y cells (**d–f**). Compared to DT40 chicken lymphocyte WT cells, TKO cells with triple knockout of InsP_3_R were resistant to pfl-mediated peak elevation in [Ca^2+^]_c_ (**a–c**). TKO (**a–c**) or BATPA-AM at 10µM (**d**–**f**) dramatically attenuated pfl-mediated peak elevation of [Ca^2+y^]_c_ (**b** and **e**), as well as the average integrated Ca^2+^ responses calculated as the area under the curve (AUC) (**c** and **f**), as an increase above its own baseline before addition of propofol at 200 µM. Statistical analysis was performed for cytosolic Ca^2+^ level at the time of peak elevation and AUC in DT40 (**b** and **c**) or in the presence or absence of BAPTA-AM in SH-SY5Y cells (**e** and **f**). All values represent Mean ± SEM from the average of at least 30 cells measured from a minimum of four separate experiments (N ≥ 4). **** means P < 0.0001 (**b,e,f**), *** means P < 0.001 (**c**).

Similar to inhalational GAs^12–14^, propofol significantly increased [Ca^2+^]_c_ in wild type DT40 chicken lymphocytes but virtually not in DT40 cells with triple knock out of all isoforms of InsP_3_R (TKO), when measured during the peak elevation of [Ca^2+^]_c_ in WT control cells (Fig. 1a,b, *58*.*6* ± *3*.*9 vs 7*.*4* ± *0*.*5*, *P* < *0*.*0001*). The average integrated Ca^2+^ responses calculated as the area under the curve (AUC) increase above its baseline after exposure to propofol, was significantly decreased in TKO cells compared to its WT control (Fig. 1c, 109999 ± *7752 vs 37540* ± *2771*, *P* < *0*.*001*). In SH-SY5Y cells which predominantly express (99%) of InsP_3_Rs-1, propofol alone significantly elevated [Ca^2+^]_c,_ an effect that was significantly attenuated by the Ca^2+^ chelator, BAPTA-AM, at the time when [Ca^2+^]_c_ reached its peak level in the absence of BAPTA-AM (Fig. 1e, *318* ± *58 vs 140* ± *63*, *P* < *0*.*001)* and the average AUC increase above its baseline after exposure to propofol (Fig. 1f, 154375 ± *11 vs 26148* ± *4*, *P* < *0*.*0001*).

**Figure 2:**
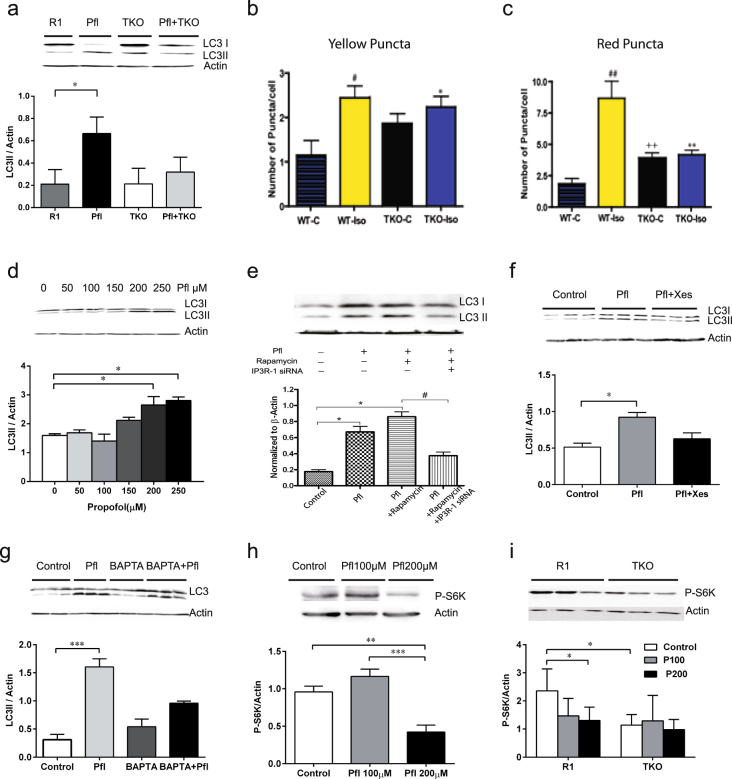
Isoflurane and propofol stimulate autophagy in an InsP_3_R- and mTOR- dependent pathway. In DT40 chicken lymphocyte cells, autophagosome biomarker LC3II protein determined by Western Blot was significantly increased under the 200 µM propofol (Pfl) treatment for 2.5h in DT40 cells with expression of only type 1 InsP_3_R (R1), but not as much in DT40 with triple knock out of InsP_3_Rs (TKO) cells (**a**). (**b**,**c**) DT40 WT or TKO cells stably overexpressing the mRFP-GFP-LC3 constructs were treated with 2.4% isoflurane (Iso) for 24 h. Baseline autophagy activity (both autophagosome and autolysosome) increased in TKO compared to WT cells. 2.4% Iso for 24 h significantly increased condensed LC3-II in both autophagosomes (**b**) and autolysosomes (**c**) in WT but not in TKO cells. Data: Mean ± SEM, one-way ANOVA followed by Dunnett’s post hoc test. *P < 0.05, **P < 0.01, ^++^P < 0.01, ^#^P < 0.05, ^##^P < 0.01 compared to wild type control (WT). In human SH-SY5Y neuroblastoma cells, propofol treatment for 2.5 hours at indicated concentrations dose-dependently increased the autophagosome biomarker LC3II (**d**), starting at a pharmacological concentration of 200 µM, and could be significantly augmented by a 30 minute pretreatment with the autophagy stimulator rapamycin at 1 µM (**e**), but inhibited by siRNA of type 1 InsP_3_R (InsP_3_R-1) (**e**), 30 min pretreatment of InsP_3_R inhibitor xestospongin C at 2 µM (**f**) and BAPTA-AM at 10 µM (**g**). Propofol dose- and InsP_3_R-1- dependently inhibited mTOR by demonstrating a proportional decrease of the mTOR enzyme product p-S6K in SH-SY5Y cells (**h**) and DT40 cells with expression of type 1 InsP_3_R but not in TKO cells (**i**). Data represent Mean ± SEM from a minimum of 2 repeats of 3 separate experiments (**d–i**, N ≥ 3), one-way ANOVA followed by Dunnett’s post hoc test. *P < 0.05, **P < 0.01, ***P < 0.001. ^#^P < 0.05.

Elevation of [Ca^2+^]_c_ via activation of InsP_3_R has been shown to release Beclin-1 from BCL-2^15^, which induces autophagy via a mTOR-dependent pathway by sequential activation of phosphorylated AMPK, inhibition of mTOR function and subsequent stimulation of autophagy^6^. As both isoflurane^7^ and propofol (Fig. 1) can activate InsP_3_Rs and raise [Ca^2+^]_c_, we examined if those commonly used GAs (isoflurane vs. propofol) affect autophagy in a mTOR-dependent pathway. In DT40 cells, propofol significantly increased levels of LC3II—a biomarker for autophagy activity and flux—in TKO cells with sole expression of type 1 InsP_3_R (InsP_3_R-1) but not in TKO DT40 cells (Fig. 2a, *P* < *0*.*05*). We then compared changes in LC3II in both autophagosomes and autolysosomes by using a mRFP-GFP-LC3 construct and transfection procedure described previously^16^ in WT or TKO DT40 cells after treatment of 2.4% isoflurane for 24 h. Triple knockout of InsP_3_Rs (TKO) showed an increase in baseline autophagosomes (Fig. 2b, *yellow puncta*) and autolysosomes (Fig. 2c, *red puncta*), similar to previous reports on the mTOR-independent pathway model^17^ and the results in Fig. 2a. Isoflurane significantly increased LC3II in autophagosomes (Fig. 2b, *P* < *0*.*05)* and autolysosomes (Fig. 2c, *P* < *0*.*01*) in wild type cells, but the increase was not as dramatic (*2b*) or was absent (*2c*) in TKO cells. Similar to its effects on DT40 cells, propofol dose-dependently increased LC3II (Fig. 2d) in SH-SY5Y. This change was significantly promoted by the autophagy stimulator, rapamycin (Fig. 2e, P < 0.05), but was inhibited by the InsP_3_R antagonist, xestospongin C (Fig. 2f, *0.51* ± *0.05* v*s 0*.*92* ± *0*.*06*, *P* < *0*.*05*), and siRNA of type 1 InsP_3_R (InsP_3_R-1) (Fig. 2e, *P* < *0*.*05*). To examine if elevated [Ca^2+^]_c_ (Fig. 1) is involved in propofol’s effects on autophagy, we used BAPTA-AM to blunt elevation of [Ca^2+^]_c_. BAPTA-AM significantly ameliorated elevation of LC3II induced by propofol (Fig. 2g, *0.31* ± *0.09 vs 1*.*6* ± *0*.*15*, *P* < *0*.*001*) in SH-SY5Y cells. Since the inhibition of mTOR regulates the induction of mTOR-dependent autophagy triggered by elevation of cytosolic Ca^2+^ ^6^, we further investigated the effects of GAs on mTOR activity. Propofol significantly inhibited mTOR, as evidenced by the decreased mTOR product p-S6K dose-dependently in SH-SY5Y cells (Fig. 2h, *P* < *0*.*01 or P* < *0*.*001*). This inhibition depended on the presence of InsP_3_R-1 because absence of InsP_3_R in TKO DT40 cells abolished propofol's ability to decrease p-S6K (Fig. 2i, *P* < *0*.*05*).

### Adequate autophagy is protective against propofol-induced cytotoxicity

**Figure 3:**
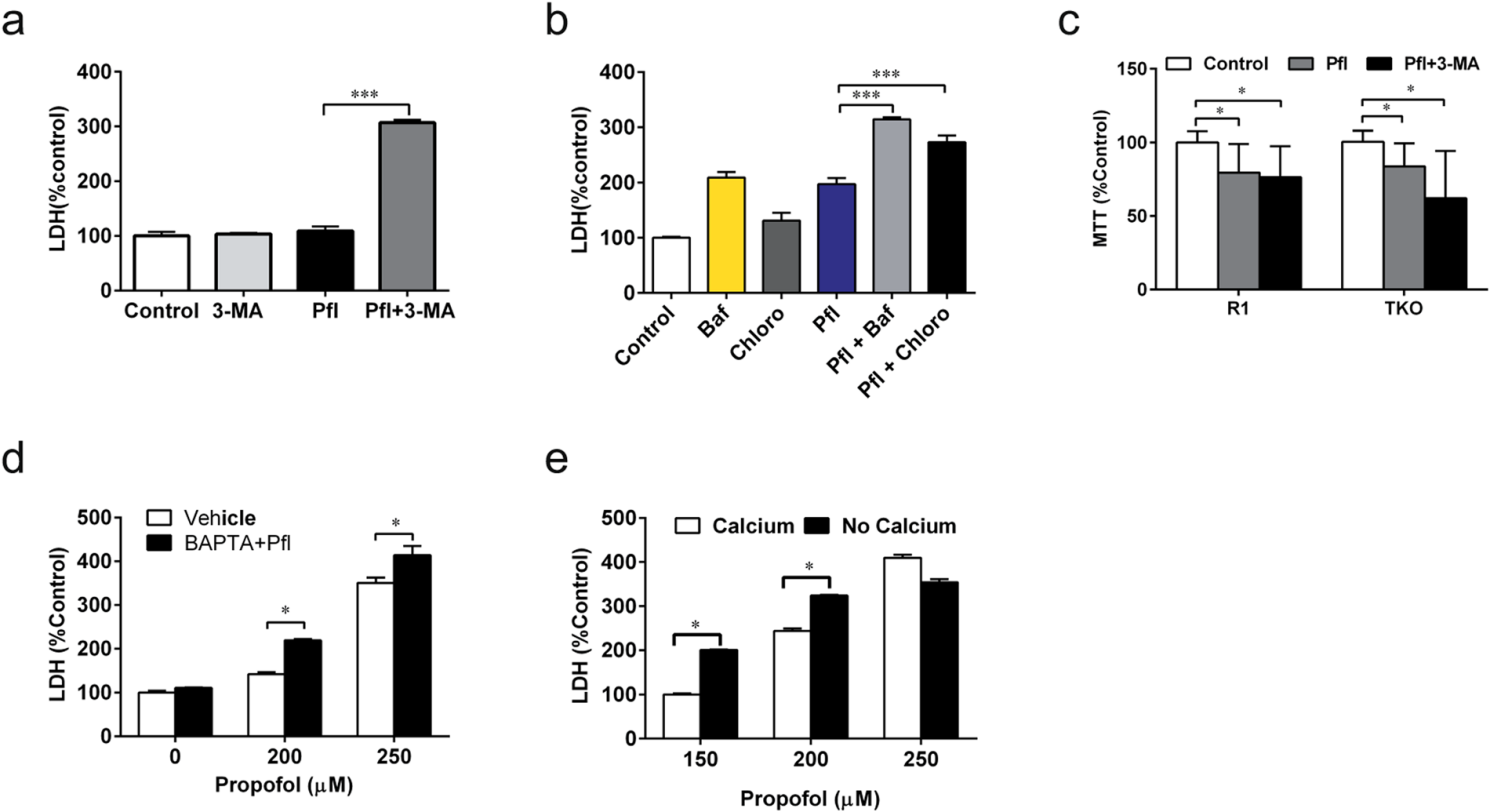
Adequate autophagy protects cells from propofol toxicity. (**a**) SH-SY5Y human neuroblastoma cells were treated with 200 µM propofol alone or together with 5mM autophagy general inhibitor 3-MA for 24h in medium without serum. (**b**) SH-SY5Y Cells were treated with 200µM propofol in the absence or presence of 40nM bafilomycin (Baf) or 10 µM chloroquine (Chloro) pretreatment for 30 min. Supernatants were collected for LDH release assay. (**c**) DT40 cells with triple knockout of InsP_3_R (TKO) or TKO with expression of recombined type 1 InsP_3_R (R1) were treated with either 200µM propofol alone or in combination with 10µl of autophagy inhibitor 3-MA for 24h. Cell viability was then measured with MTT assay. Cell damage was determined by LDH release assay in SH-SY5Y cells after treatment with propofol at various concentrations either with 30 min pretreatment of intracellular Ca^2+^ elevation inhibitor 5 µM BAPTA-AM (**d**) or in the culture medium without addition of Ca^2+^ to maximally inhibit Ca^2+^ influx (**e**). Data represent Mean ± SEM from at least four separate experiments (N ≥ 4), one-way ANOVA followed by Dunnett’s post hoc test. *P < 0.05, ***P < 0.001.

Because autophagy activity plays an essential role in determining cell survival fate^18^, we explored the effects of GAs on cell death via autophagy. Previous studies have suggested that adequate autophagy is cytoprotective against various types of detrimental stresses^19^. Although propofol caused dose-dependent cytotoxicity in SH-SY5Y cells, general inhibition of autophagy by 3-MA significantly increased this propofol cytotoxicity (Fig. 3a, *P* < *0*.*001*). On the other hand, impairment of autophagy flux may be cytotoxic and promote cell death when induced by various stress^20^. Bafilomycin and chloroquine both further significantly increased propofol-induced neuronal injury or death in SH-SY5Y cells, possibly by impairing autophagy flux (Fig. 3b, *P* < *0*.*05*, *P* < *0*.*001*). We further demonstrated that adequate propofol exposure may stimulate autophagy via activation of InsP_3_R-1 and provide cytoprotection, as the autophagy inhibitor 3-MA could only potentiate propofol-induced cytotoxicity in DT40 TKO cells but not in TKO cells with expression of type 1 InsP_3_R (Fig. 3c, *P* < *0*.*05*).

Taking this result one step further, we determined the role of [Ca^2+^]_c_ elevation in propofol cytotoxicity. Buffering the elevation of [Ca^2+^]_c_ by BAPTA-AM significantly potentiated propofol-induced cytotoxicity (Fig. 3d, *P* < *0*.*05*), which could be attributed to deficient autophagy stimulation by adequately elevated [Ca^2+^]_c_ (Fig. 1). Finally, to examine the role of Ca^2+^ influx from the extracellular space in propofol cytotoxicity, we used media depleted of Ca^2+^ during propofol treatment. Similar to BAPTA-AM, inhibition of Ca^2+^ influx potentiated propofol-induced toxicity, possibly by inhibiting the physiological autophagy stimulated by adequate elevation of [Ca^2+^]_c_ (Fig. 3e, *P* < *0*.*05*).

### Excessive autophagy or impairment of autophagy flux promotes GA-mediated cytotoxicity. 

**Figure 4:**
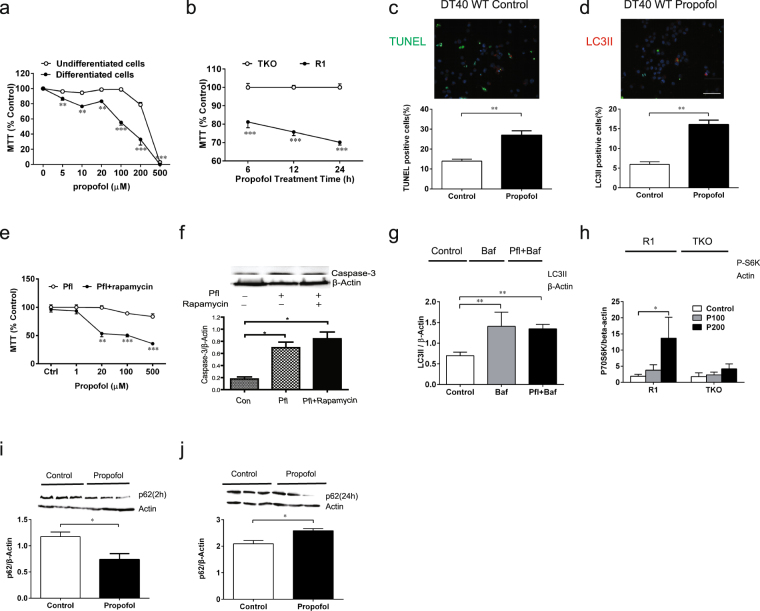
Anesthetic-induced cell death was associated with anesthetics’ regulatory effects on autophagy activity via activation of InsP_3_Rs. (**a**) In SH-SY5Y human neuroblastoma cells, propofol treatment at various concentrations for 24 h induced cell damage (MTT reduction) in a dose-dependent manner, with differentiated SH-SY5Y neurons displaying more sensitivity to propofol toxicity, even at clinically relevant concentrations (up to 20 µM). (**b**) In DT40 chicken lymphocytes with triple knock out of InsP_3_R (TKO) or TKO with expression of recombinant type 1 InsP_3_R (R1), treatment with 200 µM propofol time-dependently induced cell death, as represented by reduced MTT in R1 but not TKO cells. (**c**) Propofol at 50 µM for 2 h induced apoptosis in DT40 WT, showing significantly increased TUNEL positive cells and the parallel elevation of the autophagy activity biomarker LC3II (**d**) Scale bar=100µM (c and d). (**e**) Autophagy stimulator rapamycin at 1 µM significantly potentiated propofol-mediated reduction of MTT and elevation of active caspase-3 (**f**), which is representive of cell damage by apoptosis. (**g**) Bafilomycin (Baf), an inhibitor of the autolysosome formation stage of autophagy maturation, did not increase LC3II induced by propofol further, suggesting propofol-mediated impairment of autophagy flux from autophagosomes to autolysosomes. (**h**) DT40 TKO or R1 cells were treated with propofol at 100 (P100) or 200 (P200) µM for 24h before being collected for western with anti P-S6K. i) In SH-SY5Y human neuroblastoma cells, a short incubation of 200 µM propofol (2 h) trended to increase while prolonged 200 µM propofol exposure (24 **h**,**j**) trended to decrease P62 degradation measured by Western Blot, indicating dual effects of propofol for both increasing autophagy activity at short exposure and impairing autophagy flux from autophagosome to autolysosome at prolonged exposure. Data represents Means ± SEM from a minimum of 4 separate experiments (**a**–**j**). One-way (**f,g**,**j**) or two-way (**a**,**d**,**e**) ANOVA followed by Dunnett’s post hoc test or student t test (**c**,**d**,**i**,**j**) *P < 0.05; **P < 0.01, ***P < 0.001 compared to corresponding control respectively.

When SH-SY5Y cells were differentiated into neurons, they were much more sensitive to propofol-induced cytotoxicity, as measured by MTT release assay, even at concentrations as low as 5 µM, a clinically relevant plasma concentration, compared to non-differentiated SH-SY5Y cells (Fig. 4a, *96.2* ± *1.3 vs 86*.*9* ± *2*.*4*, *P* < *0*.*05*). Additionally, propofol-induced cytotoxicity is not only dose-dependent (Fig. 4a) but also time-dependent (Fig. 4b), which is consistent with the toxic effects of other inhalational GAs ^13^. It seems that propofol-mediated autophagy and apoptosis are closely related to each other because the biomarkers for apoptosis (TUNEL) and for autophagy stimulation or flux impairment (elevation of LC3II) increased in parallel after propofol treatment of propofol in WT DT40 cells (Fig. 4c, *13*.*9* ± *1*.*0 vs 27* ± *2*.*2*, *P* < *0*.*05 and 4d*, *5*.*9* ± *0*.*6 vs 16*.*1* ± *1*.*1*, *P* < *0*.*0001*). Similarly, excessive stimulation of autophagy by rapamycin in SH-SY5Y differentiated neurons significantly potentiated propofol-induced cytotoxicity (Fig. 4e, *P* < *0*.*01 or 0*.*001*) and apoptosis (Fig. 4f, *P* < *0*.*05*). The propofol-mediated increase of LC3-II could represent either the stimulation of autophagy or impairment of autophagy flux, especially the conversion of autophagosomes to autolysosomes.

We further determined that propofol at high concentrations (200 µM, Fig. 4g) elevated LC3-II with concomitant use of bafilomycin, an inhibitor of lysosome function and autolysosome formation^21^ and an agent capable of differentiating the causes of LC3II elevation. Bafilomycin is a relatively selective agent that impairs autophagy flux by itself. If propofol increases LC3-II by stimulation of autophagy induction, addition of bafilomycin will increase LC3-II further more. Otherwise it will indicate that propofol impair autophagy flux. Propofol did not further increase LC_3_II even in the presence of bafilomycin (Fig. 4g, *P* < *0*.*01*), suggesting that propofol at a high concentration (200µM) for a prolonged duration (24 h) increased LC3-II by impairment of autophagy flux rather than induction of autophagy activity, further worsening propofol-mediated apoptosis (Fig. 4c).

In contrast to its stimulatory effect on autophagy through inhibition of mTOR after short exposure (Fig. 2h and i, 2.5 hr), propofol at the same concentration (200 µM) for a prolonged exposure (24 h) inhibited autophagy induction by increasing activation of mTOR via activation of InsP_3_R, as evidenced by the increased mTOR product p70-56 k only in DT40-R1 but not in TKO cells (Fig. 4h, *P* < *0*.*05*). These results suggest that excessive propofol exposure may have negative feedback activation on mTOR function and can inhibit autophagy induction via over-activation of InsP_3_R-1.

p62 is an ubiquitin- and LC3-binding protein that is degraded by the autophagy-lysosome pathway and is accumulated during deficient autophagy or during impaired autophagy flux^22^. Similar to its dual effects on mTOR activity (Figs 2h and i vs. 4h), propofol at 200 µM for 2 or 24 h affected the p62 level differentially, decreasing at short exposure but increasing at prolonged exposure (Fig. 4i *1.17* ± *0.15 vs 0*.*74* ± *0*.*19*, *P* < *0*.*05* and 4*j*, *2*.*1* ± *0*.*21 vs 2*.*6* ± *0*.*13*, *P* < *0*.*05*). Therefore short exposure of propofol increased autophagy stimulation via a mTOR-dependent pathway (Fig. 2h and i) and promoted autophagy flux, providing cytoprotection (Fig. 3), while prolonged propofol exposure impaired autophagy flux (Fig. 4g and h) and thus decreased the turnover of p62 via the autophagy-lysosome pathway (Fig. 4j), inducing cytotoxicity.

### Anesthetics impaired autophagy by disruption of mitochondria and lysosome function

**Figure 5:**
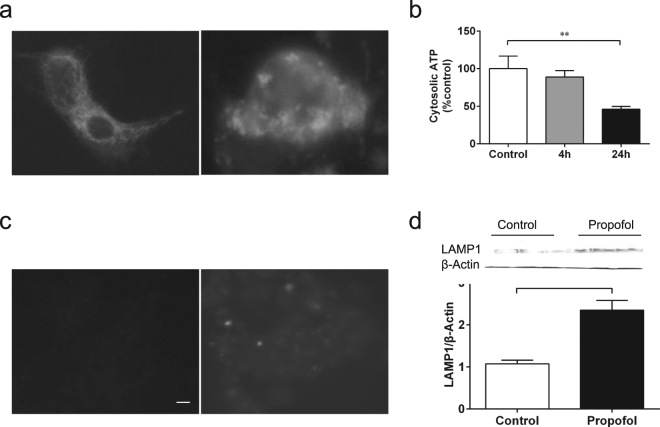
Propofol caused mitochondrial damage and elevated lysosomal pH. In SH-SY5Y human neuroblastoma cells, 200µM propofol for 24h obviously damaged mitochondria structures. (**a**) Pictures represented typical and repeatable changes of mitochondria structure in a single SH-SY5Y cell. (**b**) Prolonged exposure with 200 µM propofol for 24h, but not 4h, significantly reduced cytosolic ATP levels. (**c**) SH-SY5Y cells were treated with 200µM propofol for 5 or 24h before being stained for lysosome pH. Picture represented a typical and repeatable increase of lysosome pH. Scale bar, 10µm. (**d**) Prolonged exposure with 200 µM propofol for 24 h significantly increased lysosome protein LAMP1 expression measured by Western Blot.

As mitochondria and lysosome function are integral to autophagy^23^ and cell death^24^, we investigated if GA effects on mitochondria and lysosome function influence autophagy. In SH-SY5Y cells, propofol at prolonged treatment (24 h) obviously disrupted mitochondria structure in a single cell, as shown by mitochondria green staining (Fig. 5a). As for mitochondria function, propofol for prolonged (24 h) but not short (4 h) exposure significantly decreased ATP production, an indirect biomarker for impaired mitochondria function, in the cytosolic space (Fig. 5b, (*100* ± *16*.*4 vs 46* ± *3*.*85*, *P* < *0*.*05)*, consistent with the dual effects of propofol on cell damage (Fig. 3 vs. Figure 4) and autophagy (Fig. 4i,j). Excessive activation of InsP_3_R may deplete ER Ca^2+^ and therefore impair protein maturation inside the ER, like that of V-ATPase, a hydrogen pump that transports H^+^ from the cytosolic space into the lysosome lumen to maintain a low intraluminal pH^25^. We determined if excessive use of propofol impairs autophagy flux by disrupting lysosome function via increased pH inside the lysosome. Using lysosensor staining to monitor lysosome pH^26^, we demonstrated that propofol at prolonged exposure (24 h) but not short exposure (5 h) elevated lysosomal pH with robust reduced lysosensor signal (Fig. 5c). Eventually, the disruption of lysosome function will impair the turnover of autolysosomes and disrupt the normal function of autophagy. We can demonstrate this disruption by an increase of the lysosomal protein LAMP1, an important biomarker for autophagy flux from autophagysomes to autolysosomes^27^. Propofol at prolonged exposure significantly increased the LAMP1 protein level (Fig. 5d, *1.07* ± *0.08 vs 2*.*35* ± *0*.*23*, *P* < *0*.*05*), indicating decreased autolysosome turnover and impaired autophagy flux and providing a distinct mechanism for propofol-mediated elevation of LC3II in both tissue culture and animal studies (Fig. 4d,g,i,j,l).

### Proposed mechanism of dual effects of cytoprotection and cytotoxicity by GAs by their differential effects on autophagy via activation of InsP_3_R

**Figure 6:**
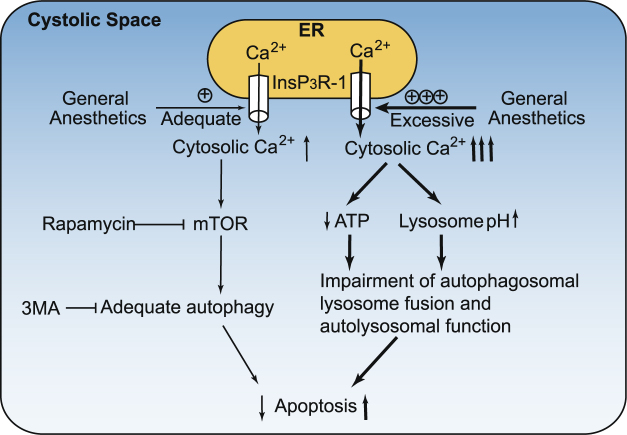
Dual effects of general anesthetics on autophagy and associated apoptosis. Moderate elevation of [Ca^2+^]_c_ by GA-mediated adequate activation of InsP_3_R receptors for a short duration may increase autophagy activity via a mTOR-dependent pathway and inhibit apoptosis (left side). Rapamycin increased while 3-MA decreased autophagy. On the other hand, abnormal elevation of [Ca^2+^]_c_ by GA-mediated excessive activation of InsP_3_Rs for a prolonged duration may result in abnormal elevation of mitochondria Ca^2+^ concentration ([Ca^2+^]_m_) and impair autophagy flux by reducing ATP production and increasing lysosomal pH and subsequently impairing lysosome and autolysosome function, which ultimately promotes apoptosis. GA: General Anesthetics.

Moderate elevation of [Ca^2+^]_c_ by short exposure of general anesthetic via adequate activation of InsP_3_Rs may increase physiological autophagy activity via a mTOR-dependent pathway (Figs 2 and 6) and provide cytoprotection (Figs 3 and 6). Inhibition of this adequate autophagy by 3-MA or by impairing adequate elevation of [Ca^2+^]_c_ may promote GA-caused cell death (Figs 3 and 6). On the other hand, prolonged use of general anesthetics at high concentrations may cause over activation of InsP_3_R and abnormal elevation of [Ca^2+^]_c_, which may result in mitochondrial overload of Ca^2+^, disruption of mitochondria structure, collapse of mitochondrial potential, and reduction of normal ATP production (Fig. 5). Over-activation of InsP_3_R and the subsequent abnormal Ca^2+^ release from the ER may impair lysosome function (Fig. 5) in the following two ways: 1). Increased pH inside the lysosome’s lumen; 2). Impairment of autophagosome and lysosome fusion, and autophagy flux, due to the abnormally high level of [Ca^2+^]_c_^28^. All these effects individually or coordinately impair autophagy flux and promote cell death or neurodegeneration (Figs 4, 5 and 6).

## Discussion

Isoflurane alone at clinically relevant concentrations activates InsP_3_R and releases Ca^2+^ from the ER, increasing cytosolic and mitochondrial Ca^2+^ concentrations^7,13,14^. Indirect evidence suggests that sevoflurane and desflurane also activate InsP_3_R^14^. Intravenous anesthetic propofol, too, can activate InsP_3_R (Fig. 1). Likely, propofol activates the isoform InsP_3_R-1 because the neuroblastoma cells (SH-SY5Y) have primarily (>99%) InsP_3_R-1 (Fig. 1b)^29^. Considering InsP_3_R-1 is the primary subtype of InsP_3_R, an essential player in the regulation of cell survival, GAs may exert their effects on cell survival via activation of InsP_3_R-1 in neurons.

Anesthetics like halothane activate the other major ER Ca^2+^ release pathway, the RyR channel complex^30^, which is thought to be the basis for the dangerous clinical occurrence of malignant hyperthermia^31^. Like InsP_3_Rs, RyRs are expressed throughout the nervous system and play important roles in both normal cell function and in neurodegenerative disease^32^. Our previous study indicated the involvement of RyR activation in isoflurane-induced apoptosis in neuronal tissue cultures^33^. Thus, both InsP_3_R and RyR contribute to regulating intracellular Ca^2+^ homeostasis and may interact with each other through a common Ca^2+^-induced Ca^2+^ release pathway in normal neuronal function and neurodegeneration.

Although general anesthetics have been shown to be both neuroprotective and neurotoxic in both *in vitro* and *in vivo* models depending on the degrees of exposure^9,34,35^, the underlining mechanisms are unclear. The proposed mechanisms for anesthetic neuroprotection include: adequate preconditioning, generation of reactive oxygen species (ROS), activation of G-protein coupled receptors, moderate elevation of [Ca^2+^]_c_, etc. The proposed mechanisms for anesthetic neurotoxicity include: excessive and abnormal activation of NMDA and GABA A receptors, generation of free radicals, activation of P^75^ growth factors, inflammation and excessive elevation of [Ca^2+^]_c_, etc.^36^. Comparing these two processes, anesthetics often use similar mechanisms to be both neuroprotective and neurotoxic, with the degree of exposure and the level of activation on different pathways serving as the determining factor^9,35^. Like ischemic preconditioning^37^, adequate exposure of GAs may induce various endogenous neuroprotective pathways and provide neuroprotection, while excessive exposure to GAs may be overstep the limit of preconditioning and stress neurons enough to cause damage.

Regulation of intracellular Ca^2+^ homeostasis and its role in cell survival is quite complex. The rise of [Ca^2+^]_c_ is required for both cytoprotection and cytotoxicity of GAs^9^. The difference between the two is the degree and duration of [Ca^2+^]_c_ elevation. Concerning the degree of elevation, previous research suggested that a moderate elevation of [Ca^2+^]_c_ (likely an increase from around 70 to 150 nM) is cytoprotective^38^ and an excessive elevation of cytosolic Ca^2+^ is cytotoxic, such as the glutamate excitotoxicity after cerebral ischemia (which is often multiple times above baseline)^39^. As for [Ca^2+^]_c_ elevation duration, short exposure may be cytoprotective and prolonged exposure may be cytotoxic^9^. While the principle governing GAs’ dual effects on neuroprotection and neurotoxicity may be clear, the exact tipping point from neuroprotection at adequate GA exposure to neurotoxicity at excessive exposure may be varied among various types of cells, animal species and patients. Nevertheless, if this theory in preclinical studies is proven to be true in patients (partial support can already be found in some retrospective clinical studies where multiple rather than single anesthesia exposure was associated with learning or developing disorders^40^, it is critically important to delineate the safe GA doses and exposure durations in different patients for future guidelines of clinical practice. For now, it is reasonable to avoid excessive anesthesia exposure whenever possible, especially in patients vulnerable to anesthesia-mediated neuronal injury or death (e.g. the developing brains of children).

Activation of InsP_3_R may either inhibit autophagy via a mTOR-independent pathway^17^ or activate autophagy via a mTOR-dependent pathway^6^. Although a few previous studies^41^ have investigated or discussed GA effects on autophagy, the exact role of GA-mediated regulation of autophagy in cell survival or death and neurodegeneration in the developing brains was far from clear, especially the effects of GAs on intracellular Ca^2+^ homeostasis. In this pioneer study, we first demonstrated that GAs can either adequately stimulate autophagy and support cell survival or pathologically impair autophagy flux and cause neuronal injury or death via differential activation of type 1 InsP_3_R (Fig. 6), providing a novel mechanism for the detailed mechanisms of GA-mediated dual effects of neuroprotection and neurotoxicity. Current experiments have demonstrated that short or adequate exposure of propofol or isoflurane promote physiological autophagy and inhibit cell death via moderate activation of type 1 InsP_3_R and elevation of cytosolic Ca^2+^ concentration. So the adequate anesthetic exposure provide cytoprotection or neuroprotection. However, prolonged use of propofol or isoflurane may impair normal autophagy flux or function via and promote cell death via over activation of InsP_3_R and abnormal elevation of cytosolic Ca^2+^ concentration. Therefore, the current study provide a molecular mechanism explaining the dual effects of neuroprotection and neurotoxicity by propofol or isoflurane, which may be representive of intravenous or inhalational general anesthetics respectively.

Previous research has suggested that various GAs may have differing potencies to cause cell injury and death^14,42^. It seems that propofol is generally less potent in causing cytotoxicity than inhalational anesthetics^43,44^. From this study, we gather that propofol needs clinically non-relevant high concentrations and durations to cause cytotoxicity or changes in autophagy activity. In contrast, isoflurane can usually cause similar changes at clinically relevant concentrations and long durations. If confirmed in animal studies and in humans, propofol at clinically used concentrations and durations may be generally less potent than isoflurane in causing neuronal injury or death.

This study is clinically important, urging the construction of strategies to minimize the use of GAs so that they do not adversely impair autophagy function and result in brain or other organ damage. Based on the tissue culture data from this study and others, the toxicity to propofol usually starting at concentrations much higher than the clinical relevant concentrations. We assume that clinically used the propofol likely provides a plasma concentration that is cytoprotective rather than toxic, although this need to be confirmed in the future clinical studies. Isoflurane seems to be more toxic than propofol as it can cause cytotoxicity at concentrations within clinical relevant range, although frequently need long duration. Clearly, more clinical studies are urgently needed to compare relative possible unwanted neurotoxic effects between propofol and commonly used inhalational anesthetics and make sure safe concentrations of general anesthetics can be used to improve safety, especially in the developing brains which may be more vulnerable to general anesthetics mediated neurodegeneration.

Previous studies have demonstrated that propofol or isoflurane can be neuroprotective against brain damage after cerebral ischemia by promoting the physiological autophagy^45–47^, which is consistent to the results from this study. This *in vitro* study, together with other studies in tissue cultures^48,49^, provides molecular mechanism of neuroprotection by general anesthetics via promoting autophagy via adequate activation of InsP_3_ and ryanodine receptors. Isoflurane and propofol have also shown to be neurotoxic by themselves in the developing brains^50–52^ but the molecular mechanism through their effects on autophagy flux is not well studied. Current *in vitro* study suggest importance of dose and duration of general anesthetics on fate of either neuroprotection or neurotoxicity via regulation of autophagy, which need further extensive studies in both animals and patients.

This study has the following limitations: 1) Most of the propofol concentrations used are pharmacological rather than clinically relevant and less valuable to be used guiding clinical practice directly. Further studies in animals and humans are needed. 2) We have only determined the major points of the mTOR-dependent autophagy pathway induced by GAs but not the detailed points (Fig. 6), but this pioneering study will inspire more research work in the area of GA-mediated regulation of autophagy and its relationship with GA-induced cell death. 3) We primarily provide a correlative connection between GA-mediated regulation of autophagy and its effects on cell survival. More selective inhibition of autophagy such as siRNA or knock out of important autophagy regulators (e.g. mTOR, Beclin-1) may be needed, rather than just 3-HK or rapamycin, to confirm the role of autophagy in GA-mediated cell death in the future studies.

In summary, our results indicate that an adequate exposure level of isoflurane or propofol stimulates physiological autophagy via an mTOR-dependent pathway and provides cytoprotection. But, an excessive use of GAs impairs autophagy flux and function and promotes neuronal injury or death. A reasonable strategy to use GAs safely is to minimize their excessive use, at either extreme concentration or duration.

## Materials and Methods

### Reagents

DMEM/F-12, RPMI1640 culture media, BAPTA-AM, Fura2-AM and B27 were obtained from Life Technologies (New York city, NY, USA). MTT (3-(4, 5-dimethyithiazol-2-yl)-2,5-diphenyl-tetrazolium bromide), propofol, rapamycin, bafilomycin, retinoic acid, 3-MA, chloroquine and TMRE were purchased from Sigma (Saint Louis, MO, USA). 30% Acrylamide solution and nitrocellulose membrane (0.45µm) were obtained from Bio-Rad (Hercules, CA, USA). LC3B rabbit polyclonal Ab (#3868), beclin-1 rabbit mAb (#3495), AMPK rabbit mAb (#2603), p-AMPK rabbit mAb (#2535), p-S6K rabbit polyclonal Ab (#9205), LAMP1 (D2D11) XP Rabbit ab (#9091), and cleaved caspase-3 rabbit polyclonal ab (#9661) were obtained from Cell Signaling (Danvers, MA, USA) and used in 1:1000 dilution. p62 mouse monoclonal antibody (#28359,1:1000) was purchased from Santa Cruz (Dallas, TX, USA). GAPDH antibody (#9484, 1:1000) was acquired from Abcam (Cambridge, MA, USA).

### Cell Culture

DT40 cells lacking the genes for all 3 isoforms of InsP_3_Rs (DT40-KO, RIKEN Cell Bank No. RCB 1467) and DT40-KO cells stably over-expressing the rat type 1 InsP_3_R (DT40-R1) were cultured as we described previously^13^. Cells were maintained in suspension culture in RPMI 1640 media containing 10% FCS, 1% chicken serum (CS), penicillin (100 units/ml), streptomycin (100 μg/ml), and glutamine (2 mM) at 37 °C in a 95% air and 5% CO2 humidified incubator. Initial suspension densities of DT40-KO and DT40-R3 were between 1 and 3 × 10^6^ cells per ml and cells were passed every 2–3 days. SH-SY5Y were cultured in DMEM/F12 media with 10%FBS, 100 units/ml penicillin, and 100 units/ml streptomycin as previously described^53^. To differentiate neurons, 0.5ml SH-SY5Y cells were seeded from a confluent T75 into a 6-well plate, and 10µM retinoic acid (RA) and B27 were added into the medium, which was changed every two days. Under those conditions, neurons could be obtained after one week^54^.

### Choice of General Anesthetics

Although isoflurane is not the most commonly used inhalational anesthetic, it is the most studied GA in the area of anesthetic-mediated neurotoxicity. Isoflurane may be more potent in causing cell damage compared to sevoflurane or desflurane^14^. Propofol is the most commonly used and most studied intravenous general anesthetic, with increasing clinical application in outpatient day surgery.

### Immunofluorescence

Cells were rinsed briefly in PBS and fixed with 2–4% formaldehyde in PBS for 10 minutes at room temperature, and were permeabilized with 0.1% Triton X-100 in PBS for 5 minutes. Cells were then rinsed with PBS three times for 5 minutes each and blocked in 5% normal (goat) donkey serum in PBS for 60 minutes. The primary antibody was diluted 1:50 in PBS and was incubated overnight at 4 °C. The cells were rinsed in PBS for 5 minutes each with high salt PBS and then rinsed two more times for 2 min. They were then incubated in secondary antibody diluted with PBS for 1 to 2 hours at RT in the dark. Lastly, the coverslips were rinsed with PBS once and stained with Hoechst 33342 1:1000 in PBS for 2–5 minutes. After being washed with PBS 3X5 min, the cells were mounted with Gold antifade reagent, cured on a flat surface in the dark overnight and sealed with nail polish.

### Cytotoxicity assays

The MTT reduction or LDH release assays determine relatively early or late stages of cell damage, respectively^13^. The LDH assay was performed using a Promaga kit CytoTox 96 Non-Radioactive Cytotoxicity Assay (Product number G1780) per manufacturer’s instructions. Cells were plated in a 24 well plate one day prior to the experiment. The first row was left blank and each treatment was done quadraplicately. After each treatment, 50 µl supernatant was collected from each well with equal amount of substrate. The collection was incubated for 30′ at room temperature in the dark and then read with a micro plate reader at 490 nm. For the MTT assay, cells were seeded in a 96 well plate. MTT was prepared using PBS to 5 mg/ml. After treatment, cells were changed into 100 µl fresh medium with 10 µl MTT per well and incubated for 4 h. The reactants were dissolved with 100 µl 10%SDS/0.01M HCl for 4 h or overnight. The results of both LDH release and MTT reduction assays were expressed as a percentage of control. TUNEL assay was performed per instructions of the manufacturer (Roche *In Situ* Cell Death Detection Kit, Fluorescein, product no 11684795910). Briefly fragmented DNA-3′-OH ends were labeled with fluorescein-dUTP in the presence of TdT enzyme. The coverslips were counterstained with DAPI.

### Western blot analysis

The cells were seeded into a 6 well plate with 2ml of cells per well. Following treatment, cells were collected and spun down. They were washed with PBS and lysed in a buffer solution containing 50mM Tris/HCl, pH7.8, 150 mM NaCl, 1% Triton X-100, 100 uM PMSF, a 1X dilution of complete protease and a phosphotase inhibitor mixture (Roche) for 5 min and vortexed. The lysates were cleared by centrifugation at 12,000rpm for 10min. Protein samples were run on 8 or 15% polyacrylamide gels and transferred onto nitrocellulose membranes. Primary antibodies were incubated overnight at 4 °C. Each independent experiment was repeated three times. Protein amount was normalized with actin. The images were quantitated with ImageJ software (http://rsb.info.nih.gov/ij/).

### Autophagy flux assay

To determine the autophagic flux (transition from autophagosome to autolysosome to lysosome), we used an mRFP-GFP-LC3 construct^16^. DT-40 were transfected with the mRFP-GFP-LC3 plasmid (Addgene, Cambridge, MA) using Lipofectamine 2000 (Invitrogen, Carlsbad, CA) according to the manufacturer’s protocol. One day after transfection, cells were exposed to 2.4% isoflurane for 24 hrs. Following isoflurane exposure, cultures were fixed and mounted on cover glasses for microscopy. Colocalized LC3 puncta in both autophagosomes (GFP+mRFP+, yellow color) and autolysosomes (GFP-mRFP+, red color) were quantified across all conditions indicated in texts or figures. Colocalization efficiency was measured with ImageJ software and all data are given as LC3-puncta per cell.

### ATP level measurement

ATP level was measured per the instructions of ATPLite Luminescence ATP Detection Assay System (Perkin-Elmer) using the modified method described previously^55^. 50 μL of mammalian cell lysis solution was added to 100 μL of cell suspension per well of a microplate. The plate was shaken and then 50 μL substrate solution was added to the wells. The luminescence was measured with a BioTech Synergy H1 plate reader.

### Mitochondria and lysosome staining

We used the methods described previously^56^. Cells were stained with 1µM MitoTracker Green (In Vitrogen) for 30′ and transferred to a phenol red free medium. TME staining was done by adding TMRE to cells in media to a final concentration of 50–400 nM and incubated for 20 minutes at 37 °C. Fluorescence was monitored and photographed using an Olympus IX70 inverted microscope and the IPLab version 3.7 Imaging Processing and Analysis software (Biovision Technologies, Exton, PA). Lysosomes were stained with 1µM LysoSensor Yellow/Blue DND-160 (Invitrogen) and imaged using a DAPI filter set.

### Measurement of Cytosolic Calcium Concentration ([Ca^2+^]_c_)

[Ca^2+^]_c_ was determined by measuring the F340/F380 ratio using Fura-2 fluorescence (Molecular probe, Eugene, OR) with a photometer coupled to an Olympus IX70 inverted microscope and the IPLab v3.7 Imaging Processing and Analysis software. The protocol to determine the F340/F380 ratio was similar to what we have described previously^12–14^ with some modifications. On the day of [Ca^2+^]_c_ measurement, the cells were loaded with 2.5 µM Fura-2AM (Molecular Probes, Eugene, OR) in Krebs-Ringer buffer for 30 min at room temperature. The cells were then placed in a sealed chamber (Warner Instruments, Hamden, CT) connected with multiple inflow infusion tubes and one outflow tube, which provided constant flow to the chamber. The cells were washed with Krebs-Ringer buffer through one inflow tube for the baseline measurement and then exposed to propofol via a separate inflow infusion tube driven by a syringe pump (Braintree Scientific, Braintree, MA). The fluorescence signals of Fura-2/AM were measured with excitation at 340nM and 380nM alternatively and emission at 510 nM for a period up to 18 min for each treatment. The F340/F380 ratio, which correlated to the level of cytosolic Ca^2+^ concentration, was constantly determined after exposing cells to propofol. The results of the F340/F380 ratio were averaged from a minimum of 30 cells in at least three separate experiments. The peak cytosolic Ca^2+^ and integrated calcium response, represented by area under curve (AUC), were determined and compared.

### Statistical Analysis

All data are expressed as the mean ± standard error of the mean (SEM). Statistical analysis was performed using Graphpad Prism 6. *P* < 0.05 was considered to indicate a statistically significant result. Each experiment was repeated at least three times. The experimental units (n) and statistical analyses used are indicated in the figures and legends.

### Data Availability

The datasets generated during and/or analysed during the current study are available from the corresponding author on reasonable request.
